# The Impact of PSMA PET/CT on Modern Prostate Cancer Management and Decision Making—The Urological Perspective

**DOI:** 10.3390/cancers15133402

**Published:** 2023-06-29

**Authors:** Azik Hoffman, Gilad E. Amiel

**Affiliations:** Department of Urology, Rambam Health Care Center, Haifa 3109601, Israel; g_amiel@rambam.health.gov.il

**Keywords:** prostate cancer, PSMA, PET CT

## Abstract

**Simple Summary:**

Prostate cancer diagnosis and treatment options have rapidly improved over the last few years. A growing number of publications have moved to front the utilization of PSMA-based PET CT in clinical practice aimed to improve urologists’ ability to stage and grade prostate cancer. While this important imaging modality was initially utilized in advanced prostate cancer treatment, recent publications support its use even earlier in the course of the disease, prior to initial treatment selection and as part of the follow-up afterward. As clinical experience with the use of PSMA PET CT grows, it slowly moves into the front row and becomes more evident in PCa diagnostic protocols and treatment decision-making. This review aims to better understand the role of this important tool in current PCa management based on current published data.

**Abstract:**

Prostate-specific membrane antigen (PSMA) PET use in prostate cancer treatment has recently become a routinely used imaging modality by urologists. New, established data regarding its performance in different stages of prostate cancer, as well as gaining clinical knowledge with new tracers, drives the need for urologists and other clinicians to improve the utilization of this tool. While the use of PSMA PET/CT is more common in metastatic disease, in which it outperforms classical imaging modalities and drives treatment decisions and adjustments, recently, it gained ground in localized prostate cancer as well, especially in high-risk disease. Still, PSMA PET/CT might reveal lesions within the prostate or possibly locoregional or metastatic disease, not always representing true cancer when utilized in earlier stages of the disease, potentially adding diagnostic burden and changing treatment decisions. As urological treatment options advance toward focal treatments in localized organ-confined prostate cancer, recent reports suggest the utilization of PSMA PET/CT in treatment planning and follow-up and even when choosing active surveillance. This review aims to reveal the current perspective of urologists regarding its daily use.

## 1. Introduction

Prostate cancer (PCa) is the most common malignancy in older men. Treatment options for intermediate and high-risk disease include active surveillance in low-grade disease, focal therapy, radical prostatectomy or radiation therapy, and for advanced disease, they include hormonal treatment with or without chemotherapy. Major advances in prostate cancer imaging and biopsy techniques have changed the landscape of clinical staging, profoundly affecting decision making. In recent years, many reports have been published on prostate-specific membrane antigens/PET in prostate cancer. Prostate-specific monoclonal antibody (PSMA) is a folate hydrolase cell surface glycoprotein that has been shown to correlate with the presence of PCa. However, in some cases, it may be expressed in cancers other than PCa or benign lesions in the prostate or other tissues. When prostate cells become malignant, PSMA is transferred into the luminal surface of the prostatic ducts, incorporates into the cell membrane, and becomes detectable. PSMA ligands are most labeled either with ^68^Ga or ^18^F. ^68^Ga-PSMA, initially utilized in metastatic prostate cancer imaging, is rapidly excreted in the urine and may obscure the prostate when evaluating localized disease. ^18^F-PSMA ligands produce better tumor uptake, reduced background uptake, and improved physical spatial resolution [1]. Recently, the European Association of Nuclear Medicine reported a consensus summary supporting PSMA imaging in patients with rising PSA after radical treatment to confirm a diagnosis of metachronous oligometastatic disease and PSA persistence following treatment, and while still debatable, recent results of prospective trials support its use for the initial staging of high-risk patients [2]. The accurate staging of patients with PCa is important for therapeutic decision making. Disease relapse after surgery or radiotherapy of curative intent occurs, among other reasons, because of screen failure during staging with current diagnostic imaging techniques and missing disease extension beyond the prostate. While still not adopted routinely, PSMA PET/CT is a new whole-body scanning technique that enables the visualization of PCa with high contrast with improved diagnostic performance, mainly in metastatic disease. While current urological guidelines still adhere to bone scans and total body CT, modern urological recommendations support the use of PSMA imaging from high-risk localized prostate cancer to metastatic castrate-resistant disease [3,4,5]. With the growing use of PSMA PET/CT in different prostate cancer disease stages, a large body of evidence is changing the urological common practice of PCa management and treatment decisions, mostly preceding the guidelines. Therefore, we wish to present these changes in practice to better understand the use of this important tool in prostate cancer disease.

## 2. PSMA PET/CT in Metastatic Prostate Cancer

PSMA expression in tumor cells is progressively increased in higher-grade tumors when treated with androgen deprivation therapy, in metastatic disease, and when hormone-refractory disease develops [6,7,8]. Compared to conventional imaging, PSMA PET radiopharmaceuticals are more accurate, sensitive, and specific at identifying pelvic nodal or distant metastatic disease when used in primary staging, eventually leading to treatment modifications as reported in the ProPSMA study [9]. In this study, 152 men were randomly assigned to conventional imaging and 150 were assigned to PSMA PET/CT. Of those remaining on follow-up, 30% had pelvic nodal or distant metastatic disease. Results showed that PSMA PET/CT had a 27% (95%CI 23–31) greater accuracy than that of conventional imaging (92% [88–95] vs. 65% [60–69]; *p* < 0.0001), with higher sensitivity (85% [95%CI 74–96]) vs. 38% [95%CI 24–52]) and specificity (98% [95%CI 95–100]) vs. (91% [95%CI 85–97] in favor of PSMA PET/CT. Subgroup analyses also showed the superiority of PSMA PET-CT (32% absolute difference; 95%CI 28–35) for patients with pelvic nodal metastases and 22% absolute difference (95% vs. 74%) for patients with distant metastases. This resulted in less frequent management change in patients undergoing conventional imaging vs. PSMA PET/CT (15%, 95%CI 10–22% vs. 28%, 95%CI 21–36%, *p* = 0.008). It should also be noted that radiation exposure was higher for conventional imaging than for PSMA PET/CT. In patients who underwent second-line imaging, management change occurred in 5% of patients following conventional imaging and 27% of patients following PSMA PET/CT. However, while prostate imaging by PSMA PET/CT detects sites of prostate cancer recurrence inside and outside of the treatment area at lower PSA levels, one must take into consideration the known “Will Rogers effect” [10], potentially triggering earlier treatment initiation and raising real-life questions regarding the clinical impact of such findings on its impact on disease-specific and overall survival rather than other disease pathway timepoints [11].

PSMA overexpression is also present when the prostate cancer cell becomes castrate-resistant (CRPC). While classic CRPC treatments approved by the FDA in the past decade did not require PSMA PET/CT imaging in the staging protocol, current treatment heavily relies on this imaging modality, rendering additional stage shifting and earlier treatment initiation. The utilization of PSMA PET is currently well established in both hormone-sensitive (HSPCa) and castrate-resistant (CRPCa) advanced prostate cancer; however, it is only recommended by guidelines as an expert opinion [4,5,12,13]. The American (AUA) guidelines do acknowledge the fact that novel PET tracers appear to show greater sensitivity than conventional imaging for the detection of prostate cancer recurrence and metastases at low PSA values (<2.0 ng/mL); however, while these advanced imaging modalities may enhance the detection of metastatic lesions, the impact on treatment decisions and patients’ overall survival has not been demonstrated yet.

Recent data support a threshold of SUVmax 5.3 for ^68^Ga-PSMA-11-PET imaging as a predictor of a metastatic prostate cancer lesion [14]. Since a possible treatment for advanced disease involves adding ^177^Lu-PSMA-617 radionuclide therapy for PSMA-avid disease, prior PSMA-based imaging seems to be in place. Van der Sar et al. [15] reported their experience in utilizing ^68^Ga-PSMA-11-PET imaging in 32 CRPC patients receiving two cycles of Lu-PSMA-617 treatment. Although the exact uptake indicating sufficient tracer accumulation is still a matter of debate when a lesion-level analysis was performed, the cut-off values for a minimum specificity of 0.8 were SUV peak 12.7 (sensitivity = 0.44) and SUVmax 15.4 (sensitivity = 0.49), and 80% of non-responding metastatic lesions had lower uptake values. The results in this study indicate that tracer accumulation within a lesion (SUVpeak > 14.87 or SUVmax > 19.08) can predict an imaging-based response to Lu-PSMA-617 treatment. On a patient-level analysis, this response was highly associated with biochemical response indicated by PSA reduction following treatment [15]. Interestingly, in 10 patients (17%), no radiological or clinical response was noted (non-responders) despite sufficient tracer uptake compared to the liver tissue, indicating that PSMA uptake is not always translated into a clinical response. Therefore, the eligibility for ^177^Lu-PSMA-617 treatment based on PSMA uptake should be better defined, and the growing clinical data in this area will allow clinicians to better select patients eligible for this treatment.

Some studies quantify the whole-body PSMA tumor volume (PSMA-TV) as the sum of all PSMA-avid lesions with a fixed threshold value of SUV ≥ 3, manually and semiautomatically excluding physiological uptake sites, to be used as a predictor of metastatic disease [16,17,18,19]. While those thresholds are currently used by some clinical practitioners and urologists, they remain prone to reader bias due to false-positive PSMA uptake and experience. Interestingly, Kim et al. [20] showed that the use of different semiautomatic PET-derived tumor volume (PSMA-TV) models in patients with advanced prostate cancer predicts overall survival when comparing low and high-volume whole-body uptake. Incorporating similar thresholds into clinical data produces a nomogram predicting similar overall survival outcomes in low and high-volume diseases [21], supporting the future use of such models to further affect clinical practice.

When discussing PET-based imaging for prostate cancer, it is important to mention ^18^F-FDG-based imaging. Although ^18^F-FDG has relatively low diagnostic accuracy in metastatic prostate cancer compared to other malignancies, it has some potential benefits in CRPC patients who are under long-term androgen deprivation therapy. ADT has been reported to reduce PSMA uptake, which might reduce PET PSMA accuracy in these patients. Chen et al. evaluated the benefit of using the two tracers, ^68^Ga-PSMA and ^18^F-FDG, in 56 CRPC patients. The detection rate (75.0% vs. 51.8%, *p* = 0.004) and the number of positive lesions were higher with ^68^Ga-PSMA imaging. However, 23.2% (13/56) of patients had at least one lesion on ^18^F-FDG imaging not detected by PSMA. Positive FDG uptake was more common in patients with Gleason ≥ 8 and high PSA (>7.9 ng/mL). The authors concluded that by adding FDG PET/CT, the detection rate could be increased from 75% to 85.7% for any site of disease, mostly in patients with more pathologically aggressive and PSA-producing diseases [22].

Recent data regarding the overexpression of PSMA in advanced prostate cancer has led to a therapeutic opportunity of utilizing PSMA as radioligand targeted therapy, possibly as an additional line of treatment in these patients. A recently published review summarized the updated data regarding this therapy [23]. The alpha emitter ^223^Ra-dichloride has been shown to provide survival benefits, mainly in bone metastatic CRPC patients, and beta emitters showed some promising responses, although probably not affecting long-term response. ^225^Ac and ^213^Bi, high LET alpha emitters, were pointed out as possible treatment options to be further evaluated [23]. The use of the novel radioligand ^225^Ac-PSMA has been further reported as a second and third-line treatment of CRPC patients [24]. Overall, 91% of the patients experienced at least a 50% decline in blood PSA levels while remaining under ADT treatment, possibly predicting improvement in overall survival. While additional prospective evaluation is still warranted, PSMA-based radioligands remain a promising therapeutic opportunity in advanced prostate cancer.

## 3. PSMA PET/CT in the Detection of Prostate Cancer Bone Metastasis

Advanced stages of PCa are commonly diagnosed with bone metastases (BM). The imaging of BM is important for disease localization and characterization to direct accurate treatment. In the case of BM, it is similarly important to evaluate their size and number during follow-up and after therapy, as proper second-line therapy selection relies on it. Bone metastases are often osteoblastic but can be very heterogenic. Historically, PSA levels following treatment along with bone scan, lesion morphology on computerized tomography, and fluorine-18-fluorodeoxyglucose (^18^F-FDG) were used to determine the risk of having BM. However, combining these modalities does not always represent properly the severity of the disease. The importance of early detection of BM determines the quality of life and may lead to reduced morbidity and mortality, as bone pain due to metastatic disease affects disease prognosis [12,13]. Approved treatments for BM such as chemotherapy, highly directed external beam radiotherapy bone-modifying agents, and prostate-specific membrane antigen-targeted therapies depend on proper BM diagnosis and avoiding unnecessary treatment for bone lesions mimicking PCa BM. The sensitivity and specificity of a whole body bone scan (WBS) with SPET and SPET/CT techniques range between 96% and 94%, respectively, but PSA has been shown to predict the probability of BM detection. Retrospective analyses showed that when PSA levels are below 20 ng/mL, a positive whole-body scan is highly unlikely (NPV of 99%). Therefore, the European Association of Urology (EAU) guidelines state that a bone scan can be omitted in patients with PSA levels < 20 ng/mL and with a Gleason Score ≤ 7. The newer ^18^F-NaF PET/CT-based imaging has a high target-to-background ratio; therefore, the sensitivity and specificity for the detection of bone metastasis in high-risk PCa patients is almost 100%. In a recent publication, Pianou et al. [25] reported that a SUVmax value of PCa BM lesion was 16.57 ± 23.59 using the ^18^F-PSMA PET/CT scan. Recently, ^18^F-fluciclovine, a novel PET radiopharmaceutical, has been approved. Its main advantage is low urinary excretion and low uptake in inflammatory cells, improving the imaging within the prostatic bed in cases of biochemical relapse following radical prostatectomy [26,27]. However, larger studies are required. Additionally, ^18^F-fluciclovine is currently less available for common practice, and its current high cost affects its use.

## 4. PSMA PET/CT in Organ-Confined and Locally Advanced Prostate Cancer Treatment

The ability of PSMA PET/CT to identify intraprostatic PCa is relatively good, with a sensitivity of 87–98% and specificity of 91–96% [8,25,26,27,28,29,30]. While these results suggest a very accurate imaging modality, clearly, it may not be indicative of disease extent later in time. Since many intermediate and high-risk PCa patients planned for surgery have a larger volume and possibly harbor higher grades of disease, it is easier to detect local disease before surgery. Clinicians must also remember that while PSMA uptake is correlated with PCa pathological stage and SUVmax, a significant overlap is present. Additionally, non-tumor-related uptake is a known effect to be considered by the urologist when discussing treatment options [31]. In addition, the detection rate of all imaging modalities is influenced by prostate-specific antigen (PSA) levels, and PSMA PET/CT is no different. When PCa is initially diagnosed, imaging by PSMA PET/CT has a promising capability to improve tumor localization within the prostate, especially when combined with multiparametric MRI. Therefore, for primary staging, PSMA PET/CT is practically used in real life when intermediate and high-risk PCa is detected on biopsy following MRI imaging of the prostate as part of PCa diagnosis by MRI fusion biopsies. Some clinical centers do offer the use of PSMA PET/MRI imaging in primary localized disease, although this is limited to high and intermediate-risk patients and has not shown clear benefits compared to PSMA PET/CT. In the recurrence setting, PET/MRI can be particularly helpful when the lesions are subtle on PSMA PET/CT and might be better defined by the MRI modality. In this setting, PSMA PET/CT is superior to choline PET/CT and other conventional imaging modalities. A recently published meta-analysis [32] regarding the use of 68Ga-PSMA with radical prostatectomy reported that staging PET/CT or PET/MRI detected a regional site of cancer for 74% of patients. The detection rate for sites of recurrence was 81%, with a 50% to 53% detection rate for PSA of 0.2–0.49 ng/mL and 0.50-0.99 ng/mL. ^68^Ga-PSMA PET/CT also found prostate cancer metastatic sites in pelvic lymph nodes or distant organs in a subgroup of patients. Eight studies of staging PET/CT with histologic correlations were reported. In a patient-based analysis specifically targeting pelvic lymph node metastases, the pooled sensitivity and specificity for staging were 61% and 97%, respectively. This review concluded that ^68^Ga-PSMA PET/CT had clinical relevance to detect sites of recurrence for patients with PSA recurrence after radical prostatectomy with PSA levels less than 1.0 ng/mL [32].

Stabile et al. [33] demonstrated that a PSMA PET/CT scan may provide reliable accuracy in primary nodal staging for PCa before surgery but not to all patients. Although, they demonstrated a high negative predictive value in men with a lower risk of lymph node involvement, which might be clinically useful to reduce the number of unnecessary pelvic lymph node dissections during surgery in these patients. However, negative PSMA PET/CT cannot omit extended lymph node dissection for staging in high-risk patients.

Franklin et al. [34] evaluated the ability of preoperative mp-MRI and ^68^Ga-PSMA PET/CT scans to predict pathological outcomes and identify a subgroup with very low risk (<5%) of pathological pelvic lymph node metastasis surgery. They demonstrated that preoperative ^68^Ga-PSMA PET/CT was more sensitive in identifying pathological pelvic lymph node metastasis than 3 Tesla multiparametric MRI. However, a negative preoperative ^68^Ga-PSMA PET/CT, ISUP biopsy Grade < 5 and PI-RADS < 5 prostate multiparametric MRI, or an ISUP Grade 5 with PI-RADS < 4 on multiparametric MRI was associated with a less than 5% risk of lymph node metastases.

Eydin et al. [35] demonstrated that in patients experiencing biochemical recurrence following surgery or radiation therapy, PSA levels of >0.2 ng/mL and PSA velocity of ≥1 ng/mL/year were significantly associated with uptake on PET PSMA scans. The optimal cutoffs for pointing at positive vs. negative scans in this report were ≥0.71 ng/mL for pre-scan PSA and ≥1.22 ng/mL/yr in PSA velocity.

Luiting et al. [36] performed a literature review to assess the sensitivity and specificity of ^68^Ga-PSMA PET/CT for detecting pelvic lymph node metastases in patients with primary PCa, positive predictive value in patients with biochemical recurrence after initial curative treatment, detection rate, and the impact on treatment selection of ^68^Ga-PSMA PET/CT in patients with biochemical recurrence after radical prostatectomy. They pointed out that ^68^Ga-PSMA PET/CT had a high specificity for detecting pelvic lymph node metastases in primary PCa. In addition, ^68^Ga-PSMA PET/CT had a very high positive predictive value in identifying lymph node metastases in patients with biochemical recurrence. However, the sensitivity was only moderate. They concluded that ^68^Ga-PSMA PET is not accurate enough just yet to replace pelvic lymph node dissection. Nonetheless, in the salvage setting, ^68^Ga-PSMA PET/CT had a high detection rate and a positive impact on radiotherapy planning in patients with biochemical failure after radical prostatectomy.

Radiation therapy in an adjuvant or salvage setting is the treatment of choice in PSA recurrence following surgery. It could be curative in selected patients and delay progression in others. Several randomized trials have demonstrated the benefit of adjuvant radiation therapy in patients at high risk for relapse. Early salvage radiotherapy is now recommended by the AUA, and recent studies demonstrate improved biochemical progression-free survival and distant metastasis-free survival, mainly when utilized early (at low PSA levels) rather than later at a salvage setting. On the contrary, clinicians might delay early salvage treatment when PSA levels are very low or very slowly rising, aiming to prevent overtreatment in case of residual benign tissue. While biochemical failure is defined as two consecutive PSA levels above 0.2 ng/mL, PSMA PET/CT might detect disease recurrence at lower levels. Boreta et al. [37] reported the use of ^68^GA PET PSMA in locating recurrence sites following radical prostatectomy in 125 men with a median PSA of 0.4 ng/mL, resulting in PSMA-avid disease in 66 of 125 patients (53%). For 38% of these patients (25/66), at least one extrapelvic lesion was detected, and more than half (26%) were diagnosed with bone metastasis. Additionally, lymph node metastatic disease detectable by PSMA was found in 71% (47/66) of the patients. In total, 104 anatomic sites had PSMA-avid disease in 66 patients with positive PSMA scans, including 42% (44/104) sites located outside of salvage radiation therapy fields, and 9 patients with PSA < 0.2 ng/mL, 20% with pelvic lymph node disease detectable by PSMA scan and 1 patient with intrapelvic uptake. Boreta et al. concluded that PSMA-PET had the potential to influence salvage radiotherapy, as a significant amount of PCa could be visualized in areas not covered by standard radiation fields and therefore has an impact on clinical decision making, but a prospective evaluation is warranted, since very early detection of subclinical disease did not translate into survival benefits.

Nevertheless, initial PSMA imaging might improve local disease control following radical prostatectomy. Metz et al. [38] evaluated the ability of ^68^Ga-PSMA and 18f-choline to detect and affect outcomes in early (PSA *≤* 2 ng/mL) recurrence in 123 patients initially treated with radical prostatectomy. The primary endpoint was biochemical relapse-free survival (PSA recurrence), which was defined as a ≥0.2 ng/mL increase in PSA above the nadir and in two successive samples. The median biochemical relapse-free survival was 24.7 months in the ^68^Ga-PSMA group vs. 13.0 months in the ^18^F-choline group (*p* = 0.008). Similarly, androgen deprivation therapy-free survival was longer. In multivariate analysis, a short PSA doubling time before imaging (*p* = 0.005) and radiation with SBRT (*p* = 0.001) predicted earlier biochemical-free progression.

Despite these publications, current guidelines still advocate for the use of conventional imaging (CT, MRI, and bone scan). The practice guideline for clinicians remains general in nature, subtly worded as a recommendation to utilize novel PET/CT scans based on choline, PSMA, or fluciclovine in patients with PSA recurrence after the failure of local therapy as an alternative to conventional imaging or in the setting of negative conventional imaging, remaining only at a level of expert opinion in the AUA guidelines [12,13].

## 5. PSMA PET/CT Use in Active Surveillance and Focal Treatment for Prostate Cancer

While large-scale data regarding the use of PSMA PET/CT in PCa active surveillance and focal therapy guidance are lacking, available preliminary data might enlighten future directions.

One should keep in mind that while most metastatic, high Gleason score lesions tend to be PSMA-avid, primary cancer within the prostate is more heterogeneous. Cytawa et al. [39] demonstrated by immunohistochemistry that the location of cancer lesions in the prostate gland does not always correlate with ^68^Ga-PSMA PET/CT uptake. PET PSMA positivity is influenced by a combination of factors, such as the homogeneity and intensity of PSMA expression in tumor cells, Gleason grade, and tumor volume. The authors suggested a cutoff value of ≥90% PSMA-positive cells, which is strongly correlated with positive ^68^Ga-PSMA uptake [39]. Acknowledging this is highly important when considering active surveillance or minimally invasive treatment options aimed to avoid whole gland treatment.

Zamboglou et al. [40] aimed to perform a voxel-wise comparison of ^68^Ga-HBED-CC-PSMA PET/CT with prostate histopathology to evaluate the accuracy of ^68^Ga-HBED-CC-PSMA in detecting primary PCa. They demonstrated excellent correlations of ^68^Ga-HBED-CC-PSMA PET/CT and histopathology albeit with a small sample of eight out of nine patients (89%) and concluded that it allows a reliable and accurate detection and allocation of PCa as a basis for PET-guided focal therapies.

Schollhammer et al. [41] studied the Gastrin-Releasing Peptide Receptor (GRP-R), which is a neuropeptide receptor over-expressed by low-risk prostate cancer cells. A prospective head-to-head comparison of PSMA and GRP-R targeted imaging as part of clinical staging before surgery was performed, demonstrating that ^68^Ga-PSMA PET/CT detected 74.3% (26/35) of all tumor lesions and ^68^Ga-RM2 PET/CT detected 78.1% (25/32). ^68^Ga-PSMA uptake was higher in ISUP ≥ 4 vs. 1 (*p* < 0.0001) and ISUP ≥ 4 vs. 2 (*p* = 0.002), while median ^68^Ga-RM2 SUVmax was higher than the median ^68^Ga-PSMA SUVmax in the ISUP 2 subgroup (*p* = 0.01). They concluded that ^68^Ga-PSMA PET/CT is more useful for the detection of higher, more clinically significant (higher ISUP) tumors, and ^68^Ga-RM2 PET/CT trends toward the detection of low ISUP tumors. The combination of both may provide a stronger tool in the detection and decision making whether active surveillance, focal, or whole gland therapy is warranted.

Recently, a prospective multicenter imaging trial, the PRIMARY trial [42], reported the additive value of ^68^Ga-PSMA PET/CT to multiparametric MRI in clinically significant prostate cancer detection in men with no prior prostate biopsy. The researchers reported a direct correlation between prostate PSMA uptake and increased ISUP grade group disease, as the SUVmax increased from 2.5 to 10. Patients with an MRI lesion of PIRADS 4 or 5, and very high SUVmax (>9), or less suspicious lesions on MRI (PIRAD 2 or 3) and SUVmax > 12 had a 100% positive predictive value for clinically significant PCa. This might affect clinical practice, since while PIRAD 4 and 5 lesions are routinely targeted during prostate cancer MRI fusion biopsy, a PIRAD 3 lesion is considered equivocal and not targeted by many urologists performing MRI fusion biopsy. Adding the data obtained from PSMA PET/CT imaging improves clinically significant PCa detection rates, and it potentially might be incorporated with the PIRAD grading system, as a 5-point Likert grading system incorporating SUVmax, lesion locality, and location within the prostate could be used [43].

Similarly, Margel et al. [44] reported the performance of ^68^GA-PSMA PET/CT compared to multiparametric MRI in Pca detection during prostate biopsy. In this research, ^68^Ga-PSMA PET/CT lesions were characterized as suspicious for prostate cancer when a maximum standardized uptake value (SUVmax) of at least 2.5 was noted, according to the recommendation by Hoffman et al. [45]. For clinically significant PCa, specificity was higher for ^68^Ga-PSMA PET/MRI than multiparametric MRI (76%, 95%CI: 62–86 vs. 49%, 95%CI: 35–63, respectively; *p* = 0.001), and sensitivity was similar (88%, 95%CI: 69–98 vs. 92%, 95%CI: 74–99, respectively; *p* = 0.099). Equivocal MRI lesions (PI-RADS 3) pose a diagnostic challenge. Adding ^68^Ga-PSMA imaging to target biopsy increases the accuracy when biopsying these lesions, as specificity was also higher compared to multiparametric MRI: 86%, 95%CI: 73–95 vs. 59%, 95%CI: 43–74, respectively (*p* = 0.002). Finally, decision curve analysis showed that biopsies targeted by ^68^Ga-PSMA uptake increased the net benefit of multiparametric MRI among PI-RADS 3 lesions, while no benefit was added for PI-RADS 4 and 5 lesions targeted by MRI.

Given the current data, an interesting question was raised regarding the possibility that modern imaging incorporating PSMA PET/CT into state-of-the-art MRI-based imaging will make prostate biopsy redundant. Innovative research aimed to evaluate prospective patients undergoing radical prostatectomy without prior biopsy [46]. Surgery was offered for patients reluctant to undergo prior biopsy, as clinical criteria and common imaging suggested a high probability of harboring clinically significant disease. A subgroup of 27 patients fulfilling clinical criteria of a suspicious digital rectal examination, PSA *≥* 10 ng/mL, PI-RADS 4/5 on multiparametric MRI, and high suspicion of PCa on PSMA-PET were evaluated for pathological results after radical prostatectomy without prior biopsy. Following surgery, all patients were diagnosed with clinically significant Pca (>ISUP 1), a significantly high proportion of aggressive PCa (ISUP grade > 2: 66.7% vs. 23.5% of patients not fulfilling all criteria, *p* = 0.027), and high ratio (60.6%) of locally advanced disease. However, pathological specimens revealed a significant risk of harboring very aggressive intraprostatic or locally advanced disease (37% ISUP 4–5, 41.2% of positive lymph nodes, and 40.7% of positive surgical margins), raising the question of whether surgery was the proper initial treatment choice as well as additional ethical questions. Still, as new imaging modalities incorporating artificial intelligence with MRI, PSMA PET/CT, and novel biomarkers emerge, the future of PCa detection and accurate staging might be rapidly changing.

Additional preclinical innovations in PSMA imaging and pharmacokinetics are constantly improving tumor accumulation and tumor-to-background ratio, allowing better anatomical imaging and tumor detection [47,48,49,50]. Ongoing trials with PSMA PET/CT-directed treatment show promising results, possibly changing decision-making paradigms in prostate cancer earlier than expected [51,52,53,54].

## 6. Conclusions

**Figure 1:**
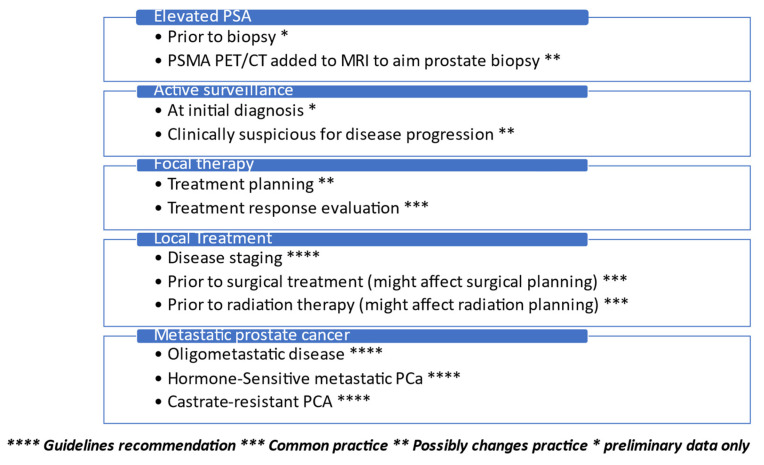
Real-life added value of PSMA PET/CT in prostate cancer treatment.

**Figure 2:**
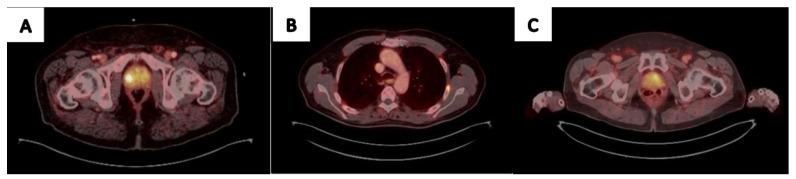
^68^Ga-PSMA Utilization in Real-Life Prostate Cancer Management: (**A**) 61 yo patient, PSA-6.1mg/ml. PIRAD 3 lesion detected in MRI. ^68^Ga-PSMA PET CT revealed SUVmax 10.6 uptake on the Rt side. Targeted biopsy detected ISUP3 PCa. (**B**) 67 yo patient, was referred for surgery due to high volume ISUP2 PCa on prostate biopsy. ^68^Ga-PSMA PET CT uptake in the Lt rib without an apparent lesion on CT, concluded as equivocal when revised. PSA remained undetectable for 1 year following surgery. (**C**) 72 yo patient, PSA 1.05 mg/ml 4.5 years following external beam radiation therapy. ^68^Ga-PSMA PET/CT imaging revealed Lt side local recurrence. Targeted biopsy detected ISUP3 PCa.

The utilization of ^68^Ga-PSMA PET/CT is constantly expanding. New agents are up and coming for various stages of the disease, and eventually, they may replace the need for multiparametric MRI before a local intervention, affect the need for lymph node dissection and its extent during surgery, determine the extent of radiation therapy, direct focal therapy planning, and probably will become the mainstay in follow-up protocols for low-risk prostate cancer. As currently utilized in metastatic disease, it is expected to play a significant role in the earlier stages of prostate cancer. We suggest a current real-world approach for clinicians to utilize PSMA imaging in prostate cancer management (Figure 1). A common utilization of PSMA PET/CT imaging based on data presented is demonstrated in Figure 2. While a growing number of publications is available, a cautious stepwise approach utilizing prospective randomized trials is the best way to assure the adoption of PSMA PET/CT in earlier stages of the disease by the urologic community.
